# Pharmacokinetics and metabolism of artemisinin (ART) in *Plasmodium yoelii*: ART-heme adduct as a potential biomarker for its resistance

**DOI:** 10.1016/j.ijpddr.2025.100603

**Published:** 2025-07-12

**Authors:** Shanshan Du, Kun Xu, Zhaohua Liu, Jie Xing

**Affiliations:** a School of Pharmaceutical Sciences, Shandong University, Jinan, China; b The Model Animal Research Center, Cheeloo College of Medicine, Shandong University, Jinan, China

**Keywords:** Artemisinin, Heme adduct, Biomarker, Resistance, Antimalarial activity

## Abstract

**Background:**

*Plasmodium falciparum* in Southeast Asia and Africa is developing resistance to the antimalarial drug artemisinin (ART). In this study, the metabolite of ART in *P. yoelii* parasites was evaluated as a potential biomarker for its antimalarial activity as well as its resistance.

**Methods:**

The induced strain of *P. yoelii* (i*Py*) was first established after long-time pressure of ART in *P. yoelii* (*Py*)-infected mice. The metabolic and pharmacokinetic profiles of ART were then studied in both *P. yoelii* parasites and infected mice. The pharmacokinetic-pharmacodynamic behaviors of ART in two strains of *P. yoelii* (*Py* and i*Py*) were compared. The pharmacokinetic parameters (*e.g*., AUC and C_max_) of ART metabolite in parasites were normalized by infected RBC (iRBC) burden.

**Results:**

Lower antimalarial activity was found for ART against i*Py* than *Py*, in terms of the 90 % growth inhibitory dose (ED_90_, 2.9-fold). In contrast with *Py*, mice infected with i*Py* could survive for at least 28 days. When ART was orally given to (i)*Py*-infected mice, ART was detected in parasites as ART-heme adduct. The plasma clearance of ART was not affected by (i)*Py*-infection, and higher plasma clearance of ART (by 3-4-fold) was found after multiple doses. After being normalized by iRBC, the exposure of ART-heme in *P. yoelii* parasites was dose-dependent, and its maximum concentration (C_max_) was reached at 3–5 h. Compared with *Py* parasites, lower iRBC-normalized exposure of ART-heme (AUC_0-t, normalized_) was found in i*Py* parasites (61.1 % of *Py* parasites) after an oral dose of ART to infected mice.

**Conclusions:**

Plasma ART concentration merely reflected drug exposure in the host. ART-heme adduct was the major metabolite for ART in *P. yoelii* parasites, and it could be a potential biomarker for the antimalarial activity of ART as well as its resistance.

## Highlights

• An artemisinin (ART)-resistant strain of *P. yoelii* was established using a murine model.
• The major metabolite of ART in *P. yoelii* parasites was ART-heme adduct.
• Plasma ART was just an indicator for the ART amount received by mice.
• ART-heme in parasites was a potential biomarker for ART antimalarial activity and resistance.

## Graphical abstract

Fig. Artemisinin (ART)-heme adduct in *Plasmodium yoelii* as a potential biomarker for artemisinin resistance.

**Figure:**
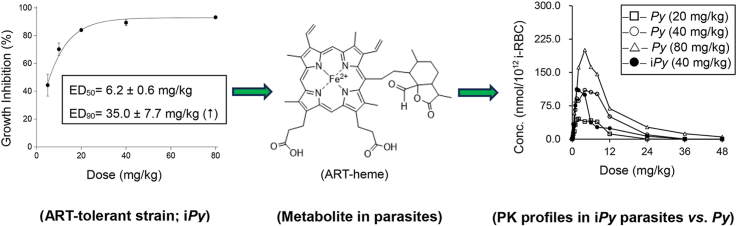

## 1 Introduction

Artemisinin (ART; Fig. S1) and its derivatives (*e.g.*, dihydroartemisinin; Fig. S1) are widely used for the treatment of uncomplicated *Plasmodium falciparum* (WHO, 2023). However, *Plasmodium falciparum* in Southeast Asia and Africa is developing resistance to ART drugs (White and Chotivanich, 2024; Rosenthal et al., 2024), with a longer clearance half-time (>5 h) after ART drug-based combination treatment. The delayed clearance of parasites is correlated with mutations in the *Pf*K13 gene (Birnbaum et al., 2020). To avoid the rapid spread of ART resistance, the full understanding of the action mechanism of ART drugs is necessary.

The structure-activity relationship showed that the endoperoxide bridge is essential for the antiplasmodial potency of ART (Rudrapal and Chetia, 2016). The underlying action mechanism of ART drugs is heme-mediated decomposition of the endoperoxide group leading to carbon-centered free radicals, which can alkylate parasite proteins (Ismail et al., 2016). These radicals can also alkylate heme itself to generate ART-heme adducts (Fig. S1), which were detected in the spleens of *Plasmodium*-infected mice treated with ART (Robert et al., 2005). Such adducts were also observed in *P. falciparum* parasites incubated *in vitro* (Ma et al., 2021), and it was revealed as an irreversible inhibitor of heme crystallization. Much lower MS abundance of ART-heme was found for ART resistance strain (DCP Cam580Y) at specific sampling time-points (Heller et al., 2018), with no information on the dynamics of ART-heme. Previous studies indicated a potential role of ART-heme as a biomarker for ART resistance. However, the exact role of ART-heme adducts, as well as their formation and clearance kinetics inside parasites, were not addressed.

The metabolic and pharmacokinetic profiles of ART in hosts have been studied in detail (Zhu et al., 2024; Zang et al., 2014). ART is absorbed rapidly with a short elimination half-life (t_1/2_, ∼1–2 h) (Zhu et al., 2024). ART underwent extensive metabolism *via* hydroxylation, loss of oxygen, and subsequent glucuronidation (Zang et al., 2014). The major metabolite of ART in human was identified to be 10β-hydroxyartemisinin (Fig. S1), which displayed comparable antimalarial activity to ART (Zhu et al., 2024); whereas deoxyartemisinin lost the antimalarial activity (O'Neill, 2005). However, the metabolic profiles of ART in *Plasmodium* parasites are not clear, with no information on its pharmacokinetic profiles in parasites.

In this study, the metabolite of ART in *P. yoelii* parasites was evaluated as a potential biomarker for its antimalarial activity as well as its resistance. A mouse model infected with *P. yoelii* was used to resemble the link of host-parasite-drug. *P. yoelii* is more resistant to chloroquine than the other species (Pattaradilokrat et al., 2022), and it is relatively easy to select resistant parasites to antimalarial drugs in mice. The induced strain of *P. yoelii* (i*Py*) was first established after long-time pressure of ART in *P. yoelii* (*Py*)-infected mice. The metabolic and pharmacokinetics profiles of ART were then studied in both *P. yoelii* parasites and infected mice. The pharmacokinetic-pharmacodynamics of ART in two strains of *P. yoelii* (*Py* and i*Py*) were compared.

## 2 Materials and methods

### 2.1 Chemicals

Artemisinin (ART; purity >99.0 %) was purchased from Kunming Pharmaceutical Co. (Yunnan, China). Artemisinin-d3 (purity >98.0 %) was purchased from TRC Canada (Ontario, Canada). Chloroquine (CQ) was purchased from the National Institutes for Food and Drug Control (purity >99.0 %, Beijing, China). The ART-heme adduct and 10β-hydroxyartemisinin (M1) were prepared in our lab (purity >95.0 %) based on previous reports (Ma et al., 2021; Zhan et al., 2017), and their structures were confirmed using UV, HRMS and/or NMR.

### 2.2 Parasite strain and animal handling

The murine malaria strain *P. yoelii* BY265 (*Py*) was obtained from the Malaria Research and Reference Reagent Resource Center (MR4). Male ICR mice (29–32 g) were supplied by Beijing Vital River Laboratory Animal Technology Co., Ltd. (Grade II, Certificate No. SYXK2021-0011). The experimental protocol was approved by the University Ethics Committee (SDU-21036) and conformed to the “Principles of Laboratory Animal Care” (NIH publication no. 85-23, revised 1985). Mice were maintained at 22 ± 2 °C and 55 ± 5 % relative humidity on a 12 h light-dark cycle. Routine room/cage cleaning and sanitation was provided. Mice were fasted for 12 h before drug administration and for a further 2 h after dosing. Water was freely available during experiments. Mice were euthanized (*i.p.* 80 mg/kg, sodium pentobarbitone) when the parasitemia was higher than 70 %.

### 2.3 Establishment of the strain of i*Py*

The establishment of ART-selection strain was based on a previous report (Zheng et al., 2022). In brief, six ICR mice were selected as the parent generation. The *P. yoelii* (*Py*)-infected mice (on day 2 post-infection) was orally given escalating doses of ART (30–80 mg/kg). The parasitemia of infected mouse was assayed, and the mice with the highest parasitemia were selected as the donors of infected RBC for the next generation. *In vivo* antimalarial assay was conducted at every five generations to determine the resistance index. The strain of i*Py* at the 25th generation was used in this study.

The mice models (n = 6 for each group) infected with *Py* or i*Py* were evaluated by physiological parameters (body weight, liver/spleen weight, and organ appearance), parasitemia, mortality, histopathological examinations (HE staining of liver), and serum biochemical assay (ALT, AST, ALP, BUN, and CREA). Mice without the parasitemia on day 28 were considered fully recovered.

### 2.4 *In vivo* antimalarial assay

The antimalarial activity of ART against *Py* or i*Py* was determined according to a previous protocol (Liu et al., 2018). Chloroquine (CQ) was used as a positive model drug. Male ICR mice were treated intraperitoneally *(i.p*.) with 1 × 10^7^ red blood cells (RBCs) infected with *Py* or i*Py*. Vehicle control or ART was orally given to mice at 24, 48 and 72 h post-inoculation. The experiment was carried out using five doses with nine mice for each dose. Parasitemia was assessed by microscopic examination of Giemsa-stained blood smears on day 4 post-inoculation. The 50 % or 90 % growth inhibitory doses (ED_50_ or ED_90_) of ART was an average of three independent measurements (three mice for each dose group). ED_50_ or ED_90_ values were obtained from prism (GraphPad) software. The resistance index of i*Py* was evaluated using the ED_90_ ratio of ART against i*Py* to that against *Py*.

### 2.5 The metabolic profiles of ART in both *P. yoelii* parasites and infected mice

For metabolite profiling of ART, *P. yoelii*-infected mice (day 3 post-infection; n = 3–6 for each biological sample) were orally given a single dose of ART (40 mg/kg). Blood samples (50 μL) were collected by the jugular venipuncture at 0, 0.5, 2, 4 and 8 h post-dosing. Mice were anesthetized with 1–3 % isoflurane by using a face mask, and bile samples were collected during 0–12 h post-dosing using a catheter inserted into the bile duct. Urine and feces samples were collected during 0–12 h post-dosing using metabolic cages. Blood samples were centrifuged (4 °C, 4000 g × 5 min) to separate plasma and *i-*RBC cells. The cell pellets were washed twice with PBS, lysed with 0.5 % saponin, and then centrifuged (4 °C, 10,000 g × 10 min) to separate RBC cytoplasm and parasites. All plasma, RBC cytoplasm and parasites samples were subjected to a protein precipitation extraction process before analysis. Urine and bile samples were pretreated by solid-phase extraction, and feces samples were pretreated by ultrasonic extraction.

### 2.6 The pharmacokinetic profiles of ART in both *P. yoelii* parasites and infected mice

*Py*-infected mice (day 3 post-infection; n = 6 for each group) were orally given a single (20, 40 or 80 mg/kg) or multiple doses of ART (40 mg/kg; once daily for three consecutive days). Healthy or i*Py*-infected mice (day 3 post-infection; n = 6 for each group) were orally given a single (40 mg/kg) or multiple oral doses of ART (40 mg/kg; once daily for three consecutive days). After a single dose or the last dose of ART to mice, 40 μL of blood samples was collected from the jugular vein inserted with a polyethylene catheter (before 24 h post-dosing) or by the jugular venipuncture (from 24 h post-dosing) at 0, 0.17, 0.33, 0.67, 1, 1.5, 2, 4, 6, 8, 12, 24, 36 and 48 post-dosing. Blood samples were centrifuged (4 °C, 4000 g × 5 min) to separate plasma and *i-*RBC cells. The cell pellets were washed twice with PBS, lysed with 0.5 % saponin, and then centrifuged (4 °C, 10,000 g × 10 min) to separate parasites. Plasma and parasite samples were stored at −80 °C until analysis.

The pharmacokinetic parameters were analyzed by non-compartmental methods using the program TOPFIT 2.0 (Thomas GmbH, Langenselbold, Germany), which included the maximum plasma concentration (*C*_max_), the time-to-peak concentration (*T*_max_), the area under the plasma/parasite concentration-time curve (AUC), and the terminal elimination half-life (*t*_1/2_). The total clearance (CL/F) of ART was calculated as dose/AUC_0-∞_. To avoid the difference in the parasites development rate between infected mice, the pharmacokinetic parameters of ART-heme (*e.g*., AUC, C_max_) were normalized by infected RBC (iRBC) burden, which was calculated by parasitemia and hematocrit (0.38). The pharmacokinetic parameters of ART-heme (*e.g.*, AUC_normalized_ and C_max,_ _normalized_) were obtained.

### 2.7 The instrumental analysis

All samples for the metabolic and pharmacokinetic study were determined by liquid chromatography tandem high-resolution mass spectrometry (LC-HRMS), which included an Agilent 1100 system and a ThermoScientific LTQ-Orbitrap Velos hybrid mass spectrometer equipped with an electrospray ionization interface. The mass spectrometer employing positive ionization was calibrated allowing for mass accuracies <5 ppm in external calibration mode. The ionization voltage was 4.5 kV, and the capillary temperature was set at 320 °C. Nitrogen was used as both the sheath gas (40 units) and auxiliary gas (5 units). For metabolite profiling, the data-dependent HR-MS^2^ acquisition was triggered when the Orbitrap detected the ions on the parent ion list or the most intense ion. The resolving power was 15,000 for full HRMS scan and 7500 for the HR-MS^2^ scans.

For quantification of ART and its metabolite M1 in mice plasma, the sample pretreatment and LC-HRMS conditions referred to a previous report (Zhu et al., 2024). For quantification of ART-heme in parasites, a 25 μL of parasite pellets was mixed with 5 μL of internal standard (clarithromycin, 500 nM) and 85 μL of methanol. The parasite mixture underwent three freeze-thaw cycles using liquid nitrogen, and then was ruptured by ultrasonic treatment. After centrifugation (4 °C, 10,000 g × 15 min), aliquot (20 μL) of the supernatant was injected for LC-HRMS analysis. The analytical methods for quantification of ART and its metabolite 10β-hydroxyartemisinin (M1) in mice plasma (Fig. S2) and ART-heme in parasites (Fig. S3) were fully validated, which included specificity, linearity, accuracy and precision (Table S1 and Table S2), recovery (80.5 % for ART-heme), and stability.

### 2.8 Statistical analysis

Experimental results were expressed as mean ± SD. The statistical analysis was performed using one-way ANOVA followed by Tukey's post-hoc test with SPSS19.0 (SPSS Inc.). Comparison of the pharmacokinetic parameters (AUC and C_max_) was performed after logarithmic transformation. Statistical significance was defined as *P* < 0.05.

## 3 Results

### 3.1 The strain of i*Py*

**Fig. 1:**
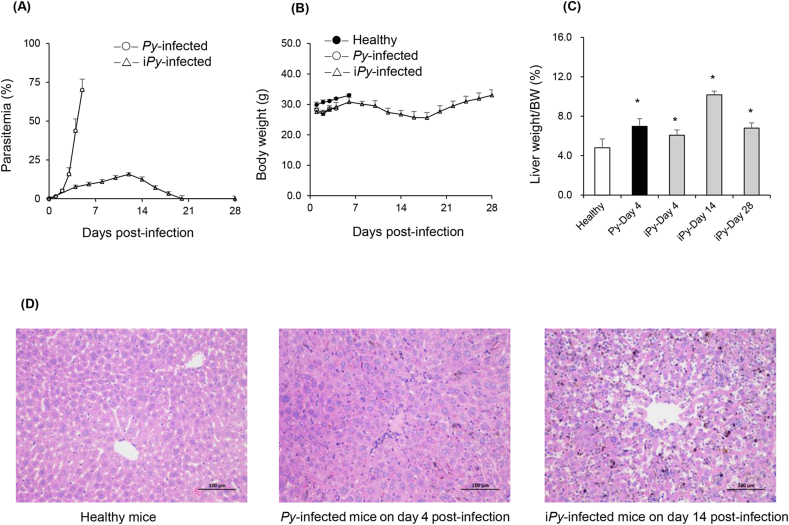
The parasitemia (A), body weight (B), the ratio of liver to body weight (C), and representative HE staining images of liver sections (D) collected from mice (n = 6 for each group) infected with *P. yoelii* (*Py*) or its induced strain (i*Py*). Values represent means ± SD. Statistical analysis was performed using one-way ANOVA followed by Tukey's Post-Hoc test. ∗*P* < 0.05, compared with healthy mice. *Py*-infected mice were euthanized on day 5 post-infection, when the parasitemia was higher than 70 %. The body weights of healthy mice were recorded for 6 days without mortality.

The *Py*-infection led to a trend of decreasing body weight of mice until being euthanized on day 5 (parasitemia >70 %), and hepatosplenomegaly was observed in mice on day 4 post-infection. Decreased weight of i*Py*-infected mice was observed until day 18 post-infection (Fig. 1), and severe hepatosplenomegaly was observed on day 14. In contrast with *Py*-infected mice (100 % mortality on day 5), all i*Py*-infected mice could survive for at least 28 days.

*Py*- or i*Py*-infected mice displayed obvious hepatotoxicity, with a significant increase (*P* < 0.05) in ALT, AST, and/or ALP on day 4 post-infection. Even higher biochemical levels (ALT and AST) were observed for i*Py*-infected mice on day 14 and recovered to almost normal levels on day 28 (Table S3). Histopathological examination of HE staining of liver sections showed cell necrosis in (i)*Py*-infected mice on day 4 or day 14 post-infection (Fig. 1), and liver recovery was found for i*Py*-infected mice on day 28 post-infection.

### 3.2 The antimalarial activity of ART against *Py* and i*Py*

The positive control CQ showed remarkable antimalarial activity against *Py* (ED_50_, 1.6 mg/kg; ED_90_, 2.8 mg/kg). ART also displayed a consistent antimalarial activity against *Py* (ED_50_, 7.2 mg/kg; ED_90_, 12.2 mg/kg). Lower antimalarial activity was observed for both ART (35.0 mg/kg) and CQ (>8.0 mg/kg) against i*Py*, in terms of ED_90_. The resistance index of i*Py* to ART was 2.9-fold, in terms of the ED_90_ ratio. The antimalarial activity of ART against *Py* or i*Py* is shown in Table 1, and the dose response curves of ART are shown in Fig. S4.

**Table 1:** Antimalarial activity of artemisinin (ART) against *plasmodium yoelii* (*Py*) and its induced strain (i*Py*).

Test drugs	*Py*		i*Py*	
	ED_50_ (mg/kg)	ED_90_ (mg/kg)	ED_50_ (mg/kg)	ED_90_ (mg/kg)
CQ	1.6 ± 0.1	2.8 ± 0.2	2.0 ± 0.1	>8.0
ART	7.2 ± 0.7	12.2 ± 1.1	6.2 ± 0.6	35.0 ± 7.7

Chloroquine (CQ) was used as a positive model drug. The experiment was performed in triplicate, and the values represent means ± standard deviation (SD).

### 3.3 Metabolite profiling of ART in *P. yoelii*-infected mice

**Fig. 2:**
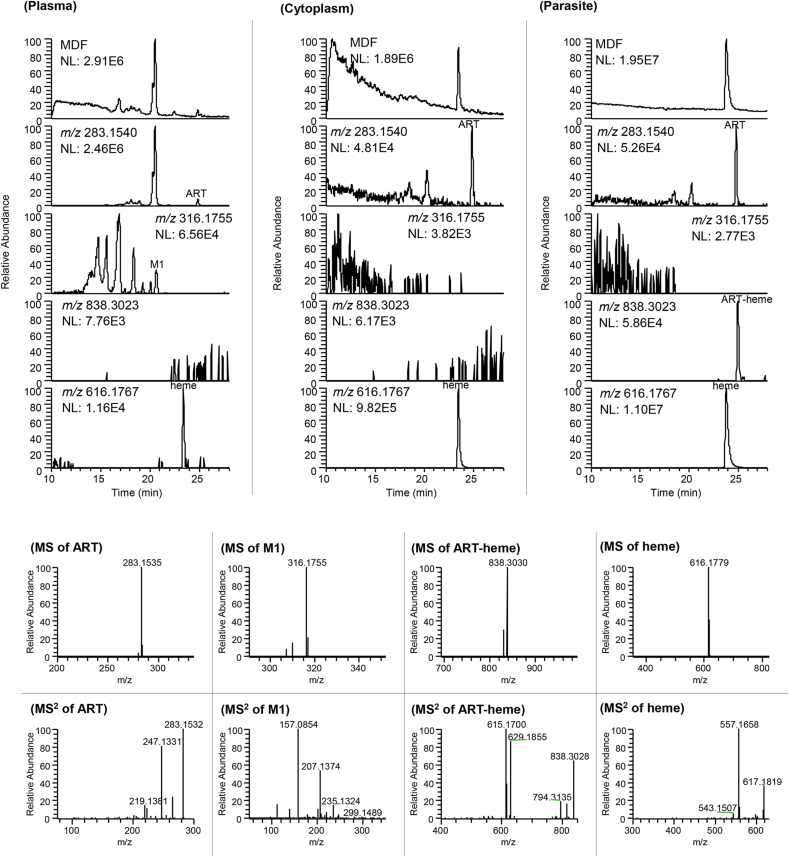
Representative selected ion chromatograms and HR-MS^n^ (n = 1–2) spectra of artemisinin (ART) and its metabolites (ART-heme adduct and M1) in a blood sample collected at 0.5 h from *Plasmodium yoelii*-infected mice (n = 6) after an oral dose of ART (40 mg/kg). MDF, mass defect filtering chromatograms. ART ([M+H]^+^, *m/z* 283.1540); M1, 10β-hydroxyartemisinin ([M + NH_4_]^+^, *m/z* 316.1755); ART-heme ([M+H]^+^, *m/z* 838.3023); heme ([M+H]^+^, *m/z* 616.1767).

The parent drug (ART) and its hydroxylated metabolite 10β-hydroxyartemisinin (M1) were detected in plasma samples of mice, with trace ART in both RBC cytoplasm and parasites (Fig. 2). A major “metabolite” detected at *m/z* 838.3023 was found for ART in (i)*Py*-parasites, with a high MS abundance of heme (Fig. 2). By comparing the HPLC behavior and HR-MS^n^ characteristics with the reference standard, the metabolite was identified as ART-heme adduct. This adduct was also detected in mice bile and feces samples, but not in urine samples after an oral dose of ART to infected mice (Fig. S5).

### 3.4 The pharmacokinetics profiles of ART in mice

**Fig. 3:**
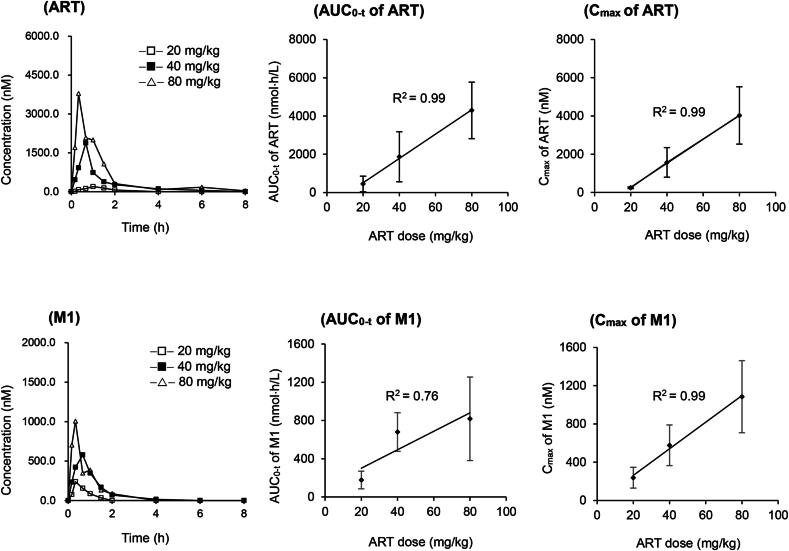
The mean plasma concentration-time profiles of artemisinin (ART) and its metabolite 10β-hydroxyartemisinin (M1) in *Plasmodium yoelii*-infected mice (n = 6 for each group) after a single dose of ART (20, 40 or 80 mg/kg), and the relationship of their pharmacokinetic parameters (AUC_0-t_ and C_max_) with ART doses. The pharmacokinetic parameters (AUC_0-t_ and C_max_) represent means ± standard deviation (SD).

After an oral dose to healthy or infected mice, ART was rapidly absorbed (T_max_, ∼0.5 h) with a short elimination half-life (t_1/2_, ∼0.9 h). The exposure of ART (AUC_0-t_ and C_max_) in *Py*-infected mice was dose-dependent (R^2^ = 0.99) within a dose range of 20–80 mg/kg (Fig. 3). The metabolic ratio (AUC_M1_/AUC_ART_) was 0.5 for ART in healthy mice after an oral dose (40 mg/kg).

The time-dependent pharmacokinetics existed for ART in both healthy and infected mice, and multiple oral doses of ART resulted in a significant (*P* < 0.05) decrease in AUC_0-t_ (∼68.0 % of single dose), accompanied with a significantly increased CL/F (by 3-4-fold). The metabolic ratio (AUC_M1_/AUC_ART_) of ART was increased after repeated dosing. The plasma pharmacokinetic profiles of ART in mice were not affected by *Py*- or i*Py*-infection (*P* > 0.05) (Table 2).

**Table 2:** The main pharmacokinetic parameters (mean ± SD) of artemisinin (ART) and its metabolite 10β-hydroxyartemisinin (M1) in healthy or infected mice (n = 6 for each group) after a single (20, 40, 80 mg/kg) or multiple oral doses of ART (40 mg/kg).

Treatment		AUC_0-t_ (μmol/L· h)	C_max_ (μmol/L)	T_max_ (h)	*t*_1/2_ (h)	CL/F (L/h/kg)	AUC_M1_/AUC_ART_
Healthy mice (40 mg/kg; Single)	ART	1.70 ± 0.14	1.57 ± 0.22	0.5 ± 0.3	0.9 ± 0.3	83.2 ± 6.2	N.A.
	M1	0.87 ± 0.12	0.75 ± 0.34	0.6 ± 0.2	0.6 ± 0.2	N.A.	0.5 ± 0.1
Healthy mice (40 mg/kg; Multiple)	ART	0.55 ± 0.32∗	0.51 ± 0.20∗	0.6 ± 0.2	0.5 ± 0.1	326.0 ± 179.4∗	N.A.
	M1	0.67 ± 0.11	0.83 ± 0.16	0.7 ± 0.0	0.5 ± 0.3	N.A.	1.5 ± 0.8
*Py*-Infected mice (20 mg/kg; Single)	ART	0.45 ± 0.41	0.24 ± 0.04	0.9 ± 0.2	0.5 ± 0.2	248.9 ± 155.1	N.A.
	M1	0.18 ± 0.09	0.24 ± 0.11	0.3 ± 0.0	0.4 ± 0.2	N.A.	0.7 ± 0.5
*Py*-Infected mice (40 mg/kg; Single)	ART	1.87 ± 1.31	1.57 ± 0.77	0.4 ± 0.3	0.9 ± 0.1	100.2 ± 55.4	N.A.
	M1	0.68 ± 0.20	0.58 ± 0.21	0.7 ± 0.0	0.3 ± 0.1	N.A.	0.4 ± 0.2
*Py*-Infected mice (80 mg/kg; Single)	ART	4.30 ± 1.48	4.02 ± 1.50	0.6 ± 0.4	0.4 ± 0.2	71.0 ± 22.2	N.A.
	M1	0.82 ± 0.44	1.08 ± 0.38	0.3 ± 0.1	0.4 ± 0.1	N.A.	0.2 ± 0.2
*Py-*Infected mice (40 mg/kg; Multiple)	ART	0.60 ± 0.24∗	0.70 ± 0.24∗	0.5 ± 0.3	0.9 ± 0.5	267.7 ± 109.5∗	N.A.
	M1	0.40 ± 0.19	0.44 ± 0.08	0.3 ± 0.1	1.0 ± 0.5	N.A.	0.7 ± 0.3
i*Py*-Infected mice (40 mg/kg; Single)	ART	1.41 ± 0.53	1.21 ± 0.57	0.4 ± 0.1	0.9 ± 0.4	110.8 ± 34.5	N.A.
	ART-M	0.37 ± 0.12	0.45 ± 0.17	0.3 ± 0.1	0.4 ± 0.1	N.A.	0.3 ± 0.1
i*Py-*Infected mice (40 mg/kg; Multiple)	ART	0.45 ± 0.31∗	0.49 ± 0.20∗	0.5 ± 0.3	0.5 ± 0.3	443.9 ± 271.3∗	N.A.
	ART-M	0.25 ± 0.16	0.29 ± 0.10	0.4 ± 0.2	0.3 ± 0.1	N.A.	0.6 ± 0.2

*Py*, *Plasmodium yoelii*; i*Py*, the induced strain of *Plasmodium yoelii*; ∗, *P* < 0.05 (single dose compared with multiple doses); N.A., not acquired. The pharmacokinetic parameters represent means ± standard deviation (SD).

### 3.5 The pharmacokinetic profiles of ART-heme in *P. yoelii* parasites

**Fig. 4:**
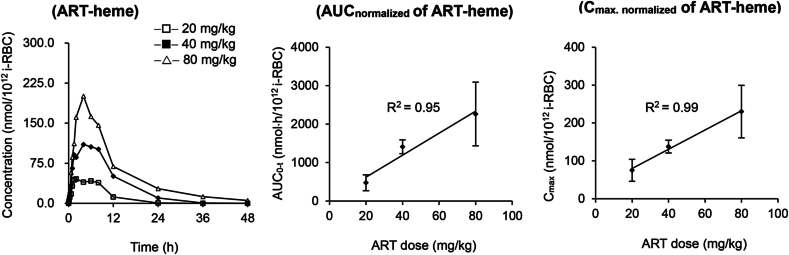
The mean iRBC-normalized concentration-time profiles of artemisinin-heme adduct (ART-heme) in *Plasmodium yoelii* parasites after a single oral dose of ART (20, 40 or 80 mg/kg) to mice (n = 6 for each group), and the relationship of its pharmacokinetic parameters (AUC_0-t, normalized_ and C_max_, _normalized_) with ART doses. The pharmacokinetic parameters (AUC_0-t, normalized_ and C_max,__normalized_) represent means ± standard deviation (SD).

The formation of ART-heme in parasites was rapid, and it was detected at 10 min after an oral dose of ART (40 mg/kg) to *Py*- or i*Py*-infected mice (Fig. 4). The C_max_ of ART-heme were reached at 3–5 h in parasites. After being normalized by iRBC burden, the exposure of ART-heme (AUC_normalized_ and C_max, normalized_) in *Py* parasites was dose-dependent (R^2^ > 0.9) within a dose range of 20–80 mg/kg (Fig. 4). Compared with a single oral dose, repeated oral dosing of ART to *Py*-infected mice led to significantly (*P* < 0.05) decreased AUC_0-t, normalized_ (57.6 %) and C_max, normalized_ (53.8 %) of ART-heme in parasites (Table 3). The iRBC-normalized exposure of ART-heme in *i*-*Py* parasites was significantly lower (*P* < 0.05) than that in *Py* parasites after an oral dose of ART (40 mg/kg) to infected mice.

**Table 3:** The main pharmacokinetic parameters (mean ± SD) of artemisinin-heme adduct (ART-heme) in *P. yoelii* (*Py*) or the induced strain of *P. yoelii* (i*Py*) parasites on day 3 post-infection to mice (n = 6 for each group) after a single (20, 40, 80 mg/kg) or multiple oral doses of ART (40 mg/kg).

	AUC_0-t, normalized_ (nmol·h/10^12^ i-RBC)	C_max, normalized_ (nmol/10^12^ i-RBC)	T_max_ (h)	*t*_1/2_ (h)
*Py*-Infected mice (20 mg/kg; Single)	475.44 ± 207.14	75.14 ± 29.09	2.8 ± 1.9	2.1 ± 0.4
*Py*-Infected mice (40 mg/kg; Single)	1412.59 ± 178.56	137.59 ± 16.99	3.8 ± 2.4	6.6 ± 1.2
*Py*-Infected mice (80 mg/kg; Single)	2263.99 ± 829.29	230.03 ± 69.29	3.9 ± 1.9	7.6 ± 3.2
*Py-*Infected mice (40 mg/kg; Multiple)	813.83 ± 255.51∗	74.04 ± 21.12∗	3.2 ± 1.8	3.8 ± 1.3
i*Py*-Infected mice (40 mg/kg; Single)	863.48 ± 425.01∗	142.65 ± 55.71	3.2 ± 1.8	5.4 ± 3.3
i*Py-*Infected mice (40 mg/kg; Multiple)	742.45 ± 151.83	281.64 ± 248.82	4.6 ± 2.2	5.1 ± 1.4

*Py*, *Plasmodium yoelii*; i*Py*, the induced strain of *Plasmodium yoelii*; i-RBC, infected red blood cell; ∗, *P* < 0.05 (compared with *Py*-infected mice after a single dose of ART at 40 mg/kg). The pharmacokinetic parameters of ART-heme in parasites were based on its iRBC-normalized concentration-time profiles, and the values represent means ± standard deviation (SD).

However, the iRBC-normalized pharmacokinetic profiles of ART-heme in i*Py* parasites did not differ (*P* > 0.05) between the single dose and multiple doses.

## 4 Discussion

The *P. falciparum* parasites are developing partial resistance to artemisinin drugs in Southeast Asia and Africa (White and Chotivanich, 2024; Rosenthal et al., 2024). However, whether the pharmacokinetic profiles of the ART drug change in ART-resistant *P. falciparum*-infected patients was not clear, not to mention the metabolic and pharmacokinetic profiles of ART drugs in parasites. In this study, the metabolic and pharmacokinetic profiles of ART were studied in both *P. yoelii*-infected mice and *P. yoelii* parasites, and its relationship with the antimalarial activity of ART as well as ART resistance was investigated. An ART-resistant strain of *P. yoelii* (i*Py*) was first established by long-time pressure of ART to *Py*-infected mice. The i*Py* on the 25th generation was selected in this study, with a resistance index of 2.9 evaluated by ED_90_ ratio of ART. *iPy* presented a multidrug resistance, with a resistance index of >2.9 for CQ. Compared with *Py*, i*Py* was not lethal to mice, which however showed reduced body weight, hepatosplenomegaly and liver injury during 18 days and then began to recover. This indicated that the virulence of *P. yoelii* was probably weakened by fitness under ART pressure.

ART was reported to accumulate in infected RBCs using the radiolabel technique (Vyas et al., 2002), which could not differentiate the parent drug with degradants. The radiolabel tag may affect the intraparasitic distribution of ART. In this study, a more specific analytical strategy was used for both metabolic and pharmacokinetic study of ART, *i.e.*, LC-HRMS. ART and its hydroxylated metabolite were detected in mice plasma, which was in correspondence with previous reports (Zhu et al., 2024). The ART-heme adduct was found to be a predominant form of ART in *P. yoelii* parasites. ART-heme adduct was probably excreted *via* bile into feces. Such adducts have been detected for ART in spleens of infected mice (Robert et al., 2005), as well as in *P. falciparum* incubated *in vitro* (Ma et al., 2021); however, the information on the formation and the clearance kinetics of ART-heme in parasites remains unknown.

The pharmacokinetic profiles of ART and its metabolites (10β-hydroxyartemisinin and ART-heme) were studied in mice infected with either *P. yoelii* (*Py*) or i*Py* after a single or multiple oral doses of ART (20–80 mg/kg), which was pharmacologically-relevant. The plasma pharmacokinetics of ART was not affected by *Py*-infection, and the exposure of ART (AUC and C_max_) in *Py*-infected mice was dose-dependent (R^2^ > 0.9), indicating that plasma ART was an indicator of the ART amount received by *Py*-infected mice. No significant difference in plasma pharmacokinetics was found for ART between *Py*- and i*Py*-infected mice, which suggested that plasma ART could not be an “indicator” of ART resistance. In accord with previous reports (Zhu et al., 2024), the time-dependent pharmacokinetics was found for ART, with a significant decrease in AUC_0-t_ after multiple doses. The formation of ART-heme was rapid, and its *T*_max_ was around 3–5 h (*vs*. 0.5 h for ART). To avoid the difference in the parasites development rate between *Py*- and i*Py*-infected mice, the pharmacokinetic parameters of ART-heme (*e.g*., AUC, C_max_) were normalized by iRBC burden**.** The iRBC-normalized exposure of ART-heme was dose-dependent (R^2^ > 0.9) in *Py* parasites, and repeated dosing could result in a decreased exposure (AUC_normalized_ and C_max, normalized_) of ART-heme. Compared with *Py*, lower AUC_0-t, normalized_ (61.1 %) was found for ART-heme in i*Py*-parasites after an oral dose of ART (40 mg/kg) to infected mice, indicating that less ART was absorbed or activated by heme in ART-resistant *Py* parasites. However, repeated dosing of ART did not lead to a significant difference in AUC_0-t, normalized_ of ART-heme in i*Py* parasites.

Several variables may exist in this study, which included inappropriate model due to species difference, non-synchronization of parasites, different resistance strain with clinical isolates. Due to genetic differences between rodent and human malaria parasites, as well as in their hosts, biological implications of such animal models must be very carefully extrapolated to human studies. Genetic profiling of the resistant strain was not accomplished in the present study. Another study showed that the relative MS abundance of the dihydroartemisinin-heme adduct was decreased in resistant *P. falciparum* incubated with dihydroartemisinin (5 μM; >500-fold of its IC_50_) *in vitro* (Heller et al., 2018), with no information on its pharmacokinetics. To resemble the parasitism between host and parasite, a mice model infected with *P. yoelii* was used in this study. Our preliminary study showed similar metabolic and pharmacokinetic profiles for ART between human and mice (not shown in detail). An ART-resistant strain was established to evaluate the potential biomarker metabolite (ART-heme) for ART resistance. An ART-resistant strain of *Plasmodium yoelii nigeriensis* was also established in another report (Robert et al., 2013), with no detectable ART-heme adduct, probably due to short sampling time (<5.5 h) and different sample pretreatment. ART-heme adduct was found not stable under acidic condition. The present analytical method for quantification of ART-heme in parasites was fully validated, which showed a satisfactory extraction recovery (80.5 %). Even though ACTs containing ART (*e.g.*, Artequick and ARCO) are commercially available, several hemisynthetic derivatives of ART (*i.e.*, dihydroartemisinin, artemether and artesunate) are more widely used in the clinic. Moreover, azaartemisnins and aminoartemisinins have been focused on over the past 30 years as antimalarial candidates (Yadav et al., 2025; Kumari et al., 2019; Rathi et al., 2024; Singh et al., 2014). The endoperoxide derivative-heme adduct as a potential biomarker for their antimalarial potency deserves further study. The underlying mechanism of reduced level of ART-heme in i*Py* parasites is another research topic, including heme availability, alteration of heme metabolism and/or ART uptake.

## 5 Conclusion

This is the first *in vivo* study demonstrating ART-heme adduct pharmacokinetics in *Plasmodium*-infected erythrocytes and its potential as a resistance biomarker. The plasma concentration of ART merely reflected drug exposure in the host.

## CRediT authorship contribution statement

**Shanshan Du:** Writing – review & editing, Methodology, Formal analysis. **Kun Xu:** Writing – review & editing, Methodology. **Zhaohua Liu:** Writing – review & editing, Methodology, Formal analysis. **Jie Xing:** Writing – review & editing, Writing – original draft, Validation, Supervision, Project administration, Funding acquisition, Formal analysis, Data curation, Conceptualization.

## Funding

This work was supported by the 10.13039/501100001809National Natural Science Foundation of China, China (no. 82274005).

## Declaration of competing interest

The authors declare no competing interests.

## List of Abbreviations

ACT, artemisinin-based combination therapy; ALT, alanine aminotransferase; ALP, alkaline phosphatase; ART, artemisinin; ART-heme, artemisinin-heme adduct; AST, aspartate aminotransferase; AUC, area under the concentrations-time curve; AUC_normalized_, area under the concentration-time curve normalized by iRBC burden; C_max_, the maximum concentration; C_max_, _normalized_, the maximum concentration normalized by iRBC; CQ, chloroquine; BUN, blood urea nitrogen; CREA, creatinine; ED_50_/ED_90_, the 50 % or 90 % growth inhibitory dose; M1, 10β-hydroxyartemisinin; LC-HRMS, liquid chromatography tandem high-resolution mass spectrometry; *Py*, *Plasmodium yoelii*; i*Py*, induced strain of *Plasmodium yoelii*; t_1/2_, terminal elimination time; CL/F, total oral clearance.

## References

[bib1] Birnbaum J., Scharf S., Schmidt S., Jonscher E., Hoeijmakers W.A.M., Flemming S., Toenhake C.G., Schmitt M., Sabitzki R., Bergmann B., Fröhlke U., Mesén-Ramírez P., Blancke Soares A., Herrmann H., Bártfai R., Spielmann T. (2020). A Kelch13-defined endocytosis pathway mediates artemisinin resistance in malaria parasites. Science.

[bib2] Heller L.E., Goggins E., Roepe P.D. (2018). Dihydroartemisinin-ferriprotoporphyrin IX adduct abundance in *Plasmodium falciparum* malarial parasites and the relationship to emerging artemisinin resistance. Biochemistry.

[bib3] Ismail H.M., Barton V.E., Panchana M., Charoensutthivarakul S., Biagini G.A., Ward S.A., O'Neill P.M. (2016). A click chemistry-based proteomic approach reveals that 1,2,4-trioxolane and artemisinin antimalarials share a common protein alkylation profile. Angew. Chem. Int. Ed. Engl..

[bib4] Kumari A., Karnatak M., Singh D., Shankar R., Jat J.L., Sharma S., Yadav D., Shrivastava R., Verma V.P. (2019). Current scenario of artemisinin and its analogues for antimalarial activity. Eur. J. Med. Chem..

[bib5] Liu H., Zhou H., Cai T., Yang A., Zang M., Xing J. (2018). Metabolism of piperaquine to its antiplasmodial metabolites and their pharmacokinetic profiles in healthy volunteers. Antimicrob. Agents Chemother..

[bib6] Ma W., Balta V.A., West R., Newlin K.N., Miljanić O.Š., Sullivan D.J., Vekilov P.G., Rimer J.D. (2021). A second mechanism employed by artemisinins to suppress *Plasmodium falciparum* hinges on inhibition of hematin crystallization. J. Biol. Chem..

[bib7] O'Neill P.M. (2005). The therapeutic potential of semi-synthetic artemisinin and synthetic endoperoxide antimalarial agents. Expet Opin. Invest. Drugs.

[bib8] Pattaradilokrat S., Wu J., Xu F., Su X.Z. (2022). The origins, isolation, and biological characterization of rodent malaria parasites. Parasitol. Int..

[bib9] Rathi K., Hassam M., Singh C., Puri S.K., Jat J.L., Prakash Verma V. (2024). Novel ether derivatives of 11-azaartemisinins with high order antimalarial activity against multidrug-resistant *Plasmodium yoelii* in Swiss mice. Bioorg. Med. Chem. Lett..

[bib10] Robert A., Benoit-Vical F., Claparols C., Meunier B. (2005). The antimalarial drug artemisinin alkylates heme in infected mice. Proc. Natl. Acad. Sci. U. S. A..

[bib11] Robert A., Claparols C., Witkowski B., Benoit-Vical F. (2013). Correlation between *Plasmodium yoelii nigeriensis* susceptibility to artemisinin and alkylation of heme by the drug. Antimicrob. Agents Chemother..

[bib12] Rosenthal P.J., Asua V., Bailey J.A., Conrad M.D., Ishengoma D.S., Kamya M.R., Rasmussen C., Tadesse F.G., Uwimana A., Fidock D.A. (2024). The emergence of artemisinin partial resistance in Africa: how do we respond?. Lancet Infect. Dis..

[bib13] Rudrapal M., Chetia D. (2016). Endoperoxide antimalarials: development, structural diversity and pharmacodynamic aspects with reference to 1,2,4-trioxane-based structural scaffold. Drug Des. Dev. Ther..

[bib14] Singh C., Verma V.P., Hassam M., Singh A.S., Naikade N.K., Puri S.K. (2014). New orally active amino- and hydroxy-functionalized 11-azaartemisinins and their derivatives with high order of antimalarial activity against multidrug-resistant *Plasmodium yoelii* in Swiss mice. J. Med. Chem..

[bib15] Vyas N., Avery B.A., Avery M.A., Wyandt C.M. (2002). Carrier-mediated partitioning of artemisinin into *Plasmodium falciparum*-infected erythrocytes. Antimicrob. Agents Chemother..

[bib16] White N.J., Chotivanich K. (2024). Artemisinin-resistant malaria. Clin. Microbiol. Rev..

[bib17] World Health Organization (2023).

[bib18] Yadav P., Rawat V., Love S.K., Verma V.P. (2025). Novel frontiers through nitrogen substitution at 6th, 10th and 11th position of artemisinin: synthetic approaches and antimalarial activity. Eur. J. Med. Chem..

[bib19] Zang M., Zhu F., Li X., Yang A., Xing J. (2014). Auto-induction of phase I and phase II metabolism of artemisinin in healthy Chinese subjects after oral administration of a new artemisinin-piperaquine fixed combination. Malar. J..

[bib20] Zhan Y., Wu Y., Xu F., Bai Y., Guan Y., Williamson J.S., Liu B. (2017). A novel dihydroxylated derivative of artemisinin from microbial transformation. Fitoterapia.

[bib21] Zheng S., Liang Y., Wang Z., Liu M., Chen Y., Ai Y., Guo W., Li G., Yuan Y., Xu Z., Wu W., Huang X., Wu Z., Xu Q., Song J., Deng C. (2022). Polymorphisms in the K13-propeller gene in artemisinin-resistant *Plasmodium* in mice. Infect. Drug Resist..

[bib22] Zhu F., Mao H., Du S., Zhou H., Zhang R., Li P., Xing J. (2024). CYP3A4-mediated metabolism of artemisinin to 10β-hydroxyartemisinin with comparable anti-malarial potency. Malar. J..

