# Supplementary material for: Pharmacokinetics and metabolism of artemisinin (ART) in Plasmodium yoelii: ART-heme adduct as a potential biomarker for its resistance
Source: Int J Parasitol Drugs Drug Resist. 2025 Jul 12;28:100603. doi: 10.1016/j.ijpddr.2025.100603 (PMC12366529; doi:10.1016/j.ijpddr.2025.100603)
Supplement: Multimedia component 1 [file mmc1.docx]

**Supporting Materials**

**Title:** Pharmacokinetics and metabolism of artemisinin (ART) in *Plasmodium yoelii*: ART-heme adduct as a potential biomarker for its resistance

Authors: Shanshan Du ^a^, Kun Xu ^a^, Zhaohua Liu ^b^, Jie Xing ^a,*^

**Journal Title:** International Journal for Parasitology: Drugs and Drug Resistance

**(M1)**

**(ART-heme)**

**(ART)**

**(Artesunate)**

**(Artemether)**

**(Dihydroartemisinin)**

**Fig. S1.** Structures of artemisinin (ART), its derivatives (dihydroartemisinin, artemether and artesunate), and its hydroxylated metabolite 10β-hydroxyartemisinin (M1) and ART-heme adduct.

**(C)**

**(B)**

**(A)**

NL: 6.29E6

*m/z* 283.1540

NL: 1.00E5

*m/z* 283.1540

NL: 4.05E3

*m/z* 283.1540

NL:2.54E5

*m/z* 316.1755

NL: 3.12E4

*m/z* 316.1755

NL: 9.70E2

*m/z* 316.1755

NL: 2.24E6

*m/z* 286.1728

NL: 2.36E6

*m/z* 286.1728

NL: 2.43E6

*m/z* 286.1728

**Fig. S2.** Representative selected ion chromatograms of (A) a blank plasma sample; (B) a blank plasma sample spiked with artemisinin (ART; 35.5 nM), 10β-hydroxyartemisinin (M1; 33.6 nM) and IS (ART-d3; 500 nM); and (C) a representative plasma sample collected at 1 h after an oral dose of ART (40 mg/kg) to *Plasmodium yoelii*-infected mice (n=6 for each group).

**(A)**

**(B)**

**(C)**

NL: 2.52E3

*m/z* 838.3023

NL: 9.75E3

*m/z* 748.4842

NL: 2.34E6

*m/z* 748.4842

NL: 2.59E6

*m/z* 748.4842

NL: 1.02E5

*m/z* 838.3023

NL: 2.02E6

*m/z* 838.3023

**Fig. S3.** Representative selected ion chromatograms of (A) a blank parasite sample; (B) a blank parasite sample spiked with artemisinin-heme adduct (ART-heme; 20 nM) and IS (clarithromycin; 500 nM); and (C) a representative parasite sample collected at 1 h after an oral dose of ART (40 mg/kg) to *Plasmodium yoelii*-infected mice (n=6 for each group).

Dose (mg/kg)

**(CQ-i*Py*)**

Growth Inhibition (%)

ED_50_= 2.0 ± 0.1 mg/kg

ED_90_= > 8.0 mg/kg

Dose (mg/kg)

**(CQ-*Py*)**

Growth Inhibition (%)

ED_50_= 1.6 ± 0.1 mg/kg

ED_90_= 2.8 ± 0.2 mg/kg

Growth Inhibition (%)

Dose (mg/kg)

**(ART-*Py*)**

ED_50_= 7.2 ± 0.7 mg/kg

ED_90_= 12.2 ± 1.1 mg/kg

Dose (mg/kg)

Growth Inhibition (%)

**(ART-i*Py*)**

ED_50_= 6.2 ± 0.6 mg/kg

ED_90_= 35.0 ± 7.7 mg/kg

**Fig. S4.** Representative dose response curves for artemisinin (ART) against *Plasmodium* *yoelii* (*Py*) or its induced strain (i*Py*). The parasite growth was measured in parasitemia units and normalized to the control values to give percentage growth inhibition. The best-fit curves and ED_50_/ED_90_ values were calculated by prism (GraphPad) software. The experiment was performed in triplicate, and the values represent means ± standard deviation (SD). Chloroquine (CQ) was used as the positive model drug.

**(Feces)**

**(Bile)**

**(Urine)**

MDF

NL: 1.77E8

MDF

NL: 9.32E7

MDF

NL: 8.29E6

*m/z* 283.1540

NL: 8.08E7

*m/z* 283.1540

NL: 2.17E7

*m/z* 283.1540

NL: 7.62E5

*m/z* 316.1755

NL: 2.87E4

*m/z* 316.1755

NL: 3.24E7

*m/z* 316.1755

NL: 1.22E7

*m/z* 838.3023

NL: 5.30E3

*m/z* 838.3023

NL: 7.12E5

*m/z* 616.1767

NL: 7.83E4

*m/z* 616.1767

NL: 2.52E6

*m/z* 838.3023

NL: 5.11E4

*m/z* 616.1767

NL: 3.65E6

**Fig. S5.** Representative selected ion chromatograms of artemisinin (ART) and its metabolites (ART-heme adduct and M1) in urine, bile or feces samples collected during 0-12 h from *Plasmodium yoelii*-infected mice (n=3-6 for each biological sample) after an oral dose of ART (40 mg/kg). MDF, mass defect filtering chromatograms. ART ([M+H]^+^, *m/z* 283.1540); M1, 10β-hydroxyartemisinin ([M+NH_4_]^+^, *m/z* 316.1755); ART-heme ([M+H]^+^, *m/z* 838.3023); heme ([M+H]^+^, *m/z* 616.1767).

**Table S1**

Accuracy and precision for quantification of artemisinin (ART) and its metabolite 10β-hydroxyartemisinin (M1) in mouse plasma using LC-HRMS (n=3 days, six replicates per day).

| Analyte | Added concentration  (nM) | Found concentration  (nM) | Accuracy  (%) | Precision (%) | |
| --- | --- | --- | --- | --- | --- |
|  |  |  |  | Intra-day | Inter-day |
| ART | 88.7 | 87.5 ± 6.4 | 98.7 | 7.2 | 6.4 |
|  | 354.6 | 338.0 ± 20.5 | 95.3 | 5.8 | 6.5 |
|  | 2127.7 | 2281.8 ± 88.7 | 107.2 | 4.2 | 3.9 |
| M1 | 83.9 | 90.0 ± 6.2 | 107.3 | 7.4 | 7.7 |
|  | 335.6 | 351.7 ± 19.4 | 104.8 | 5.8 | 5.7 |
|  | 2013.4 | 1899.4 ± 114.5 | 94.3 | 5.7 | 5.3 |

**Table S2**

Accuracy and precision for quantification of artemisinin-heme adduct (ART-heme) in *Plasmodium yoelii* parasites using LC-HRMS (n=3 days, six replicates per day).

| Added concentration  (nM) | Found concentration  (nM) | Accuracy  (%) | Precision (%) | |
| --- | --- | --- | --- | --- |
|  |  |  | Intra-day | Inter-day |
| 50.0 | 45.3 ± 2.9 | 90.6 | 5.9 | 5.1 |
| 200.0 | 197.7 ± 17.7 | 98.9 | 8.8 | 8.7 |
| 640.0 | 626.2 ± 46.5 | 97.8 | 7.3 | 7.3 |

**Table S3**

Serum biochemical parameters in mice (n=6 for each group) infected with *P. yoelii* (*Py*) or its induced strain (i*Py*) on day 4, day 14 and/or day 28 post-infection.

| Treatments | ALT (U/L) | AST (U/L) | ALP (U/L) | BUN (mM) | CREA (μM) |
| --- | --- | --- | --- | --- | --- |
| Healthy mice | 42.2 ± 3.0 | 116.2 ± 21.0 | 118.4± 22.4 | 8.1 ± 0.7 | 19.8 ± 1.4 |
| *Py*-mice (Day 4) | 117.3 ± 14.8* | 298.0 ± 42.7* | 216.7 ± 56.2* | 8.5 ± 1.0 | 15.7 ± 2.1* |
| i*Py-*mice (Day 4) | 84.2 ± 26.7* | 312.0 ± 34.4* | 160.4 ± 36.0 | 8.4 ± 0.7 | 17.2 ± 0.1* |
| i*Py-*mice (Day 14) | 224.9 ± 78.6* | 1177.2 ± 203.0* | 90.3 ± 38.0 | 11.3 ± 2.0* | 18.6 ± 4.6 |
| i*Py-*mice (Day 28) | 51.5 ± 10.2 | 184.6 ± 31.0* | 91.8 ± 7.7 | 7.6 ± 0.3 | 18.7 ± 1.0 |

*, *P* < 0.05 (compared with healthy mice). ALT, alanine aminotransferase; AST, aspartate aminotransferase; ALP, alkaline phosphatase; BUN, blood urea nitrogen; CREA, creatinine. Values represent means ± standard deviation (SD).
